# Confidence guides priority between forthcoming tasks

**DOI:** 10.1038/s41598-021-97884-2

**Published:** 2021-09-15

**Authors:** David Aguilar-Lleyda, Vincent de Gardelle

**Affiliations:** 1 Centre d’Économie de la Sorbonne, CNRS and Université Paris 1 Panthéon-Sorbonne, 112 Boulevard de l’Hôpital, 75013 Paris, France; 2 Paris School of Economics and CNRS, Paris, France; 3 Present Address: RIKEN Center for Brain Science, 2-1 Hirosawa, Wako, Saitama 351-0198 Japan

**Keywords:** Human behaviour, Decision, Perception

## Abstract

Humans can estimate confidence in their decisions, and there is increasing interest on how this feeling of confidence regulates future behavior. Here, we investigate whether confidence in a perceptual task affects prioritizing future trials of that task, independently of task performance. To do so, we experimentally dissociated confidence from performance. Participants judged whether an array of differently colored circles was closer to blue or red, and we manipulated the mean and variability of the circles’ colors across the array. We first familiarized participants with a low mean low variability condition and a high mean high variability condition, which were matched in performance despite participants being more confident in the former. Then we made participants decide in which order to complete forthcoming trials for both conditions. Crucially, prioritizing one condition was associated with being more confident in that condition compared to the other. This relationship was observed both across participants, by correlating inter-individual heterogeneity in prioritization and in confidence, and within participants, by assessing how changes in confidence with accuracy, condition and response times could predict prioritization choices. Our results suggest that confidence, above and beyond performance, guides prioritization between forthcoming tasks, strengthening the evidence for its role in regulating behavior.

## Introduction

Humans can usually evaluate the extent to which they believe their decision is correct and express this degree of belief as a confidence estimate. It has long been known that people possess this ability^1^, but the cognitive^2,3^, computational^4^ and neural^5,6^ underpinnings of confidence judgments are still being investigated. How individuals can make use of their confidence estimates to control behavior is also an important question in this topic^3,7–11^. Recent contributions to this line of research have put forward the role of confidence in learning in the absence of feedback^12–14^, monitoring errors^3,15^, comparing our performance across tasks of different nature^16,17^ or deciding how to invest our cognitive resources^18,19^.

Prioritization problems constitute yet another situation where confidence can be used to regulate behavior. Intuitively, one may expect that individuals facing a list of tasks to complete might want to tackle first the problems for which they are more confident. If there are problems for which individuals estimate they would be more likely to be successful, clearing these problems off the list first could indeed be advantageous. For instance, it would allow individuals to secure sure wins early on, thereby reducing their mental load and anxiety before tackling the more complex problems. Such preference for avoiding cognitive load and getting things done early, even at the expense of increased physical effort, has been observed in past studies and referred to as precrastination (for a review, see^20^). However, addressing high-confidence problems first could also bring potential disadvantages: although not focusing on confidence, there is evidence for physicians prioritizing easy cases negatively impacting the hospital’s revenue^21^.

The present work aims at further investigating whether confidence might affect the prioritization of simple perceptual tasks. It follows up on a recent study, where we found that confidence plays a role in the prioritization of responses about decision tasks that are already completed^22^. Indeed, when participants had completed two decision tasks, they preferred to report first the decision for which they had a higher confidence. This result could not be accounted by task difficulty (as measured by the average performance in the task) or by the accuracy of the two decisions, as we showed for instance that participants prioritized errors made with high confidence over low confidence correct responses, and that confidence affected the prioritization of responses even in trials where both difficulty and response accuracy were identical between the two decisions.

This previous study^22^ also investigated how confidence might affect the prioritization of forthcoming tasks (i.e. tasks that have yet to be completed on future stimuli). To do so, in a familiarization phase participants were presented with two tasks, one that was objectively easier (as indicated by higher average performance) and one that was objectively more difficult (as indicated by lower average performance). Then they were asked to perform new trials for both tasks in a test phase, for which they could choose the order. Participants tended to engage first with the easy task (in which presumably their confidence was higher) and leave the more difficult task for later. Although these results overall present a coherent perspective on the role of confidence as a priority signal, we must acknowledge that in these latter experiments we did not measure confidence directly and instead relied on task difficulty as a proxy for confidence. This leaves the possibility that some of these effects were not specifically due to confidence, but due to the different performance between tasks introduced by the difficulty manipulation. The goal of the present study is to fill this gap and provide further support for the independent role of confidence on prioritization of forthcoming tasks, not only by measuring confidence and linking it to prioritization, but also by experimentally dissociating confidence from performance.

**Figure 1:**
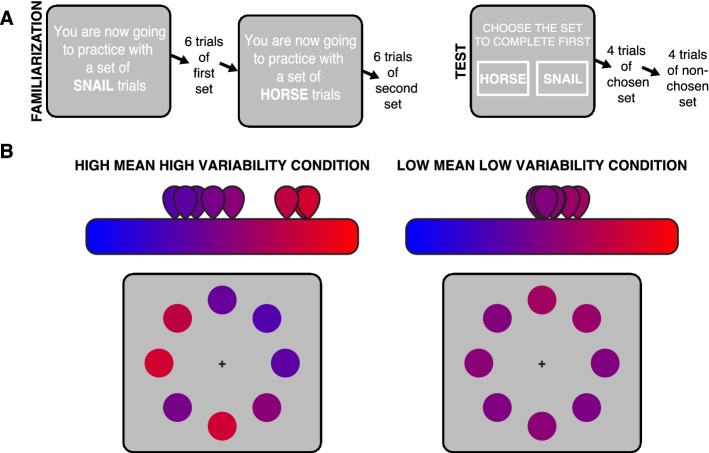
Experimental design. (**A**) Outline of a block. In the familiarization phase, participants were presented with 6 trials of two sets, with each set being introduced with an animal name. In the subsequent test phase, participants had to complete 4 trials of each of the preceding sets. However, they previously had to choose the order in which to do so, by clicking on the name of the set they wanted to complete first. (**B**) Example stimulus for each of the conditions. For both the *high mean high variability* and *low mean low variability* conditions, stimuli consisted in 8 circularly-arranged color circles. The color of each circle ranged from pure blue to pure red. Participants had to decide whether the average color across all 8 patches was closer to blue or to red. For the *high mean high variability* condition, the mean evidence generating each circle was high (i.e. further away from the midpoint between blue and red), but across-sample variability was also high. For the *low mean low variability* condition, the generative mean was of lower evidence strength (closer to the boundary), but variability was also lower.

To dissociate confidence from performance, we relied on previous studies^23–25^ showing that confidence can be biased by the variability of the evidence available for the decision, above and beyond the effect that this variability has on performance. In the present study, we adapted the familiarization/test structure mentioned above (Experiment 4 in our prior study^22^), to include a manipulation of evidence variability. On each experimental block, participants were introduced to two sets of trials and then they had to choose the order in which to complete more trials of each set (Fig. 1A). The task always involved judging whether the average color over 8 circles was closer to red or to blue (based on^26^). However, in one set of trials (*high mean high variability* condition), the average color was further away from the midpoint between red and blue and colors were more heterogeneous, while this was the opposite for the other set (*low mean low variability* condition), where the average color fell closer to the midpoint and the colors across circles were more homogenous (see Fig. 1B). By using an initial psychophysical staircase, these two conditions were matched in terms of decision performance. However, based on the aforementioned studies, we expected to find higher confidence (and therefore higher priority) for the *low mean low variability* condition. In other words, our experiment aimed at finding different subjective difficulty (i.e. different confidence) across two conditions that were equated in objective difficulty (as indicated by matched performance). To confirm that this dissociation had been produced, we collected confidence ratings for the two conditions in a separate part of the experiment.

## Methods

### Participants

A total of 29 individuals took part in the study, recruited from the database of the Laboratoire d’Économie Experimentale de Paris. They were healthy adults who reported normal or corrected-to-normal vision and provided informed consent. Participants were paid based on performance and duration (see below). The experiment complied with the declaration of Helsinki and was approved by the Paris School of Economics ethics committee. Sample size was bigger than that of the two previous studies^18,23^ that had successfully dissociated confidence from performance by the means of the present task. When recruiting participants, we followed the same guidelines as in our former^22^ study, ensuring a comparable sample size, particularly with Experiment 4, which also investigated prioritization based on confidence in forthcoming tasks.

### Apparatus and stimuli

The experiment was run using MATLAB Psychtoolbox ^27–29^ on a computer screen (17′, 1024 × 768 pixel resolution, 60 Hz refresh rate) viewed from approximately 60 cm. Stimuli appeared on a grey background.

On each of the trials of our experiment, participants had to categorize the average color of a set of 8 circles as more red or blue. Trials started with a black fixation cross presented for 400 ms, followed by 8 circles presented simultaneously for 200 ms. Each circle was uniformly colored in RGB space as [255*t, 0, 255*(1 − t)] with a color parameter t taking values between 0 (full blue) and 1 (full red). The circles, each with an area of 2500 pixels, were regularly spaced on an imaginary circle 120 pixels from fixation. After the stimulus disappeared, the fixation cross turned white and participants had to make a response. For all parts of the experiment, within each block of trials, red was the dominant component (and thus the correct response) for 50% of the trials and blue for the other 50%. Participants had to use the E and R keys of the keyboard to indicate whether the average color was closer to red or to blue. The mapping between each of the two keys and each of the two color responses was randomized across participants.

On each trial, the color parameter t for each circle was sampled from a standard normal distribution, then multiplied with the standard deviation of the corresponding condition (high: SD = 0.20, low: SD = 0.05) and then added to the mean of the corresponding condition, which depended on the specific part of the experiment.

### Procedure

The experiment started with 20 easy training trials that acquainted participants with the structure of each trial. After each response, feedback on their performance was given by displaying the message ‘CORRECT’ or ‘ERROR’.

After the training trials, participants were told that they were going to do a second, longer training session. In fact, in this session the generative mean of the stimulus parameter t was updated according to a staircase procedure, to obtain 75% accuracy in what in the main part of the experiment would be the *high mean high variability* condition and the *low mean low variability condition*. We used a one-up/one-down procedure with unequal up and down steps. The starting value for the mean was 0.75 and it was reduced by 0.0078 after a correct response and increased by 0.032 after an error. This mean parameter determined the signal strength towards red, and on a random half of the trials we used 1-t to obtain blue trials. This staircase procedure was conducted separately for the *high mean high variability* condition (SD = 0.2) and for the *low mean low variability* condition (SD = 0.05) in two interleaved staircases of 100 trials each. The decision of leaving the SD stable and updating the mean followed previous use of similar tasks^18,23^. When the staircase phase was finished, we estimated for each condition the value leading to an expected performance of 75%, from the psychometric curve linking the probability of responding ‘red’ to the value of the mean parameter. When fitting the psychometric curve to the data, we assumed that participants had no bias towards red or blue. Importantly, during this part feedback on performance was still given after each trial in order for participants to learn where the midpoint between red and blue laid. Feedback was removed for the subsequent parts of the experiment.

The main part of the experiment then started. Each of its 32 blocks was composed by a familiarization and a test phase, echoing the idea of Experiment 4 in^22^. In the familiarization phase, participants were introduced to two sets of trials: for one set, the generative mean and variance of evidence strength were high (*high mean high variability* condition), whereas for the other both mean and SD were low (*low mean low variability* condition). However, the expected performance of both sets was always 75%, with the means leading to such performance estimated from the previous staircase trials. Participants were not told about these particularities, and each set was only labeled with an animal name (randomly chosen without replacement from a predefined list of common names, see supplementary material). The design did not include a condition with high mean and low variability or a condition with low mean and high variability, because those conditions would not lead to equated performance.

After 6 trials of each set were completed, participants had to do the test phase: completing 4 more trials of each set, with these test trials determining payoff at the end of the experiment. Critically, they could choose the order in which to complete the two sets in the test phase. A screen was presented with two horizontally aligned boxes containing (in a random position) the animal names of the two sets, participants clicked on the name of the set they wanted to start with and completed the test trials in the chosen order. Animal names were used to avoid order biases generated by other labels (set A vs B, set 1 vs 2).

After the main part of this experiment, participants completed an additional 8 blocks of 24 trials where confidence was also required after each perceptual decision. Within each block, 12 trials of the *high mean high variability* condition and 12 trials of the *low mean low variability* condition were randomly interleaved. Confidence was given by clicking on a vertical scale. As the participant moved a cursor over it, an integer was displayed on the left, ranging from 50 at the bottom of the scale (meaning total uncertainty with regards to whether the choice had been correct) to 100 at the top (meaning total certainty). The question appearing on the screen was ‘To what extent are you sure that your choice was correct?’ The response was framed as a percentage: the response integer was accompanied by the ‘%’ symbol, and the scale always displayed ‘50%’ and ‘100%’ as labels on each endpoint. Confidence was only required in this final part, to avoid inducing demand effects on participants’ priority choices in the previous part.

For those parts of the experiments organized in blocks, participants were given 10 s of rest between block and block. Halfway across the staircase trials, and every four blocks in the main part, participants could take self-timed breaks. The whole experiment typically lasted around 75 min.

Participants were given a fixed 5€ plus a variable payment that could add up to an extra 16€. For the main part, for each of the 32 blocks, a trial was chosen randomly. If the response for that trial had been correct 0.5€ were awarded, and 0€ otherwise. This payoff scheme ensures participants keep a stable performance. For the confidence trials, we chose a random trial for each of the 8 blocks. Then, a number N was drawn from a uniform distribution between 0 and 100. If the number was lower than the reported confidence, payoff depended on performance as before, otherwise it depended on a random draw of a lottery with a success probability equal to N/100. If the lottery was successful 0.5€ was given, and 0€ otherwise. This scheme incentivizes participants to perform well and to judge their confidence accurately^30^. Participants were informed about payoff rules before the start of the experiment. However, despite the aforementioned payoff scheme, we made sure participants won at least 15€.

## Results

### Equal performance in the high mean high variability and low mean low variability conditions

**Figure 2:**
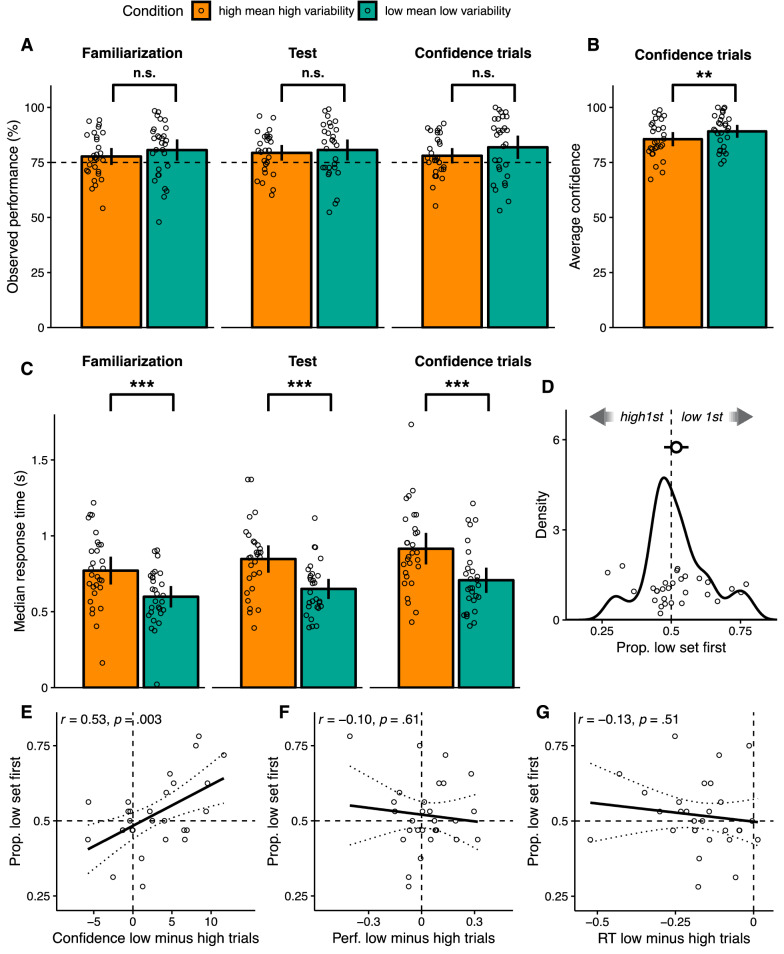
Results. (**A**) For the familiarization and test phases of the main part, and for the final trials where confidence reports were required, observed performance as a function of expected performance, in percentage, and split by condition. Bars depict across-participant averages and error bars represent 95% confidence intervals. Dots show individual participants. The horizontal dashed line marks where a hypothetical 75% observed performance would lay. (**B**) For each condition, average confidence ratings across participants. Error bars represent 95% confidence intervals. Dots show individual participants. Although confidence ratings ranged from 50 to 100, here the y-axis is scaled from 0 to 100 for easier comparison to (**A**). (**C**) For the familiarization and test phases of the main part, and for the final trials where confidence reports were required, response times, in seconds, split by condition. Dots represent the median response time of each individual participant, while bars represent averages across individual medians, with error bars denoting 95% confidence intervals. For A through C, lines connecting pairs of columns are accompanied by symbols representing *p* values for the corresponding paired t-tests described in the main text (n.s.: *p* ≥ 0.05; *: 0.05 > *p* ≥ 0.01; **: 0.01 > *p* ≥ 0.001; ***: *p* < 0.001). (**D**) Density of the proportion of blocks where participants decided to complete first the the set with trials belonging to the *low mean low variability* condition. The density uses each participant’s data. The big dot depicts the across-participant average, with error bars representing 95% confidence intervals. The dashed line corresponds to *low mean low variability* condition and *high mean high variability* condition sets being chosen first to the same extent. (**E**) Proportion of blocks where participants decided to complete first the set with trials belonging to the *low mean low variability* condition, as a function of the difference between the average confidence for *low mean low variability* condition trials minus the average confidence for *high mean high variability* condition trials. Dots depict individual participants. Solid and dotted lines correspond to across-participant linear regressions and their respective 95% normal confidence intervals. The plot does not depict the participant removed from the analyses. (**F**) Proportion of blocks where participants decided to complete first the the set with trials belonging to the *low mean low variability* condition, as a function of the difference, for the familiarization phase, between the average performance for *low mean low variability* condition trials minus the average performance for *high mean high variability* condition trials. Dots depict individual participants. Solid and dotted lines correspond to across-participant linear regressions and their respective 95% normal confidence intervals. (**G**) Proportion of blocks where participants decided to complete first the the set with trials belonging to the *low mean low variability* condition, as a function of the difference, for the familiarization phase, between the median response time for *low mean low variability* condition trials minus the median response time for *high mean high variability* condition trials. Dots depict individual participants. Solid and dotted lines correspond to across-participant linear regressions and their respective 95% normal confidence intervals. For D through G, low refers to the *low mean low variability* condition, whereas high refers to the *high mean high variability* condition.

The objective of our experimental design was to create two conditions that were matched in objective performance, but differed in confidence. To check whether such dissociation happened, we started by looking at the difference in objective performance between conditions. A paired t-test determined no significant differences in performance, neither for either phase of the main part (familiarization phase, mean performance for *high mean high variability* condition = 0.78, mean for *low mean low variability* condition = 0.81, t(28) = 1.03, *p* = 0.31, 95% CI [-0.09, 0.03], *d* = 0.19, Bayes factor for no difference between conditions = 3.14); test phase, mean for *high mean high variability* condition = 0.79, mean for *low mean low variability* condition = 0.81, (t(28) = 0.47, *p* = 0.64, 95% CI [− 0.07, 0.04], *d* = 0.09, Bayes factor for no difference between conditions = 4.58), nor for the final trials where confidence was rated (mean for *high mean high variability* condition = 0.79, mean for *low mean low variability* condition = 0.82, t(28) = 1.28, *p* = 0.21, 95% CI [− 0.10, 0.02], *d* = 0.24, Bayes factor for no difference between conditions = 2.42, see also Fig. 2A). Moreover, as developed in the supplementary material, performances in the different parts of the experiments were positively correlated across participants.

### Higher confidence in the low mean low variability condition

We then looked at the between-condition difference in those trials where confidence was rated. A paired t-test confirmed that participants were more confident in the *low mean low variability* condition (means 89.13 and 85.58, t(28) = 3.15, *p* = 0.004, 95% CI [− 5.86, − 1.24], *d* = 0.59, Bayes factor for difference between conditions = 10.30, see Fig. 2B), as found in previous studies with similar stimuli^18,23^. Taken together, the results from our within-participant analyses for performance and confidence suggest that we achieved the attempted dissociation between these two variables.

### Faster response times for the low mean low variability condition

In experimental studies, confidence is often approximated by response times, with faster responses usually being made with greater confidence. We checked whether, across the different parts of our experiment, individual median response times for the *low mean low variability* condition were faster than for the *high mean high variability* condition. Paired t-tests determined that this was the case, both for the familiarization phase (mean response time 0.60 and 0.78, t(28) = 7.55, *p* < 0.001, 95% CI [0.13, 0.22], *d* = 1.40, Bayes factor for difference between conditions = 442,301.23) and test phase (mean response time 0.65 and 0.85, t(28) = 5.65, *p* < 0.001, 95% CI [0.13, 0.27], *d* = 1.10, Bayes factor for difference between conditions = 4251.85, see Fig. 2C) of the main part and for the trials where confidence reports were requested (mean response time 0.71 and 0.92, t(28) = 5.59, *p* < 0.001, 95% CI [0.13, 0.29], *d* = 1.04, Bayes factor for difference between conditions = 3596.85). Thus, response times were shorter for the *low mean low variability* condition, consistent with the finding of higher confidence for that condition.

### Relationship between prioritizing the low mean low variability condition and confidence

Capitalizing on this dissociation between confidence and performance, we then looked for evidence for the role of confidence on priority. In our data, an obvious way to do so would be to find a general tendency for participants to prioritize the *low mean low variability* condition in the test phase. Contrary to our expectations, we could not find this effect at the group level: the proportion of blocks where the *low mean low variability* condition set was chosen first was not different from 0.5 across participants (mean = 0.52, t-test: t(28) = 0.85, *p* = 0.40, 95% CI [− 0.03, 0.06], *d* = 0.16, Bayes factor for difference between conditions = 0.27, Fig. 2D). However, a clear relation between confidence and priority emerged when we examined inter-individual differences. For each participant, we calculated the difference in average confidence for the *low mean low variability* condition minus the *high mean high variability* condition. We then correlated, across participants, this difference in confidence with the tendency to prioritize the *low mean low variability* condition set. As shown in Fig. 2D, the more participants expressed greater confidence in the *low mean low variability* condition (relative to the *high mean high variability* condition), the more they prioritized the *low mean low variability* set (*r* = 0.53, *p* = 0.003). Note that this analysis was conducted after removing one participant who always prioritized the first presented set, irrespectively of the condition, and for whom no other factor influencing priority could be evaluated (no participant showed the extreme opposite pattern of always prioritizing the last presented set). In sum, this analysis revealed that, across participants, a relationship existed indeed between confidence and priority.

### Ruling out performance and response time as drivers of priority

It could be that, although we tried to match performance between conditions, for some participants performance for one condition was higher than for the other. One could argue that this difference in performance, and not a difference in confidence, may have determined priority. To address this concern, we tested whether the difference of average performance in the familiarization phase between *low mean low variability* and *high mean high variability* condition trials was correlated across participants with the tendency to prioritize the *low mean low variability* condition set. This was not the case (*r* = − 0.10, *p* = 0.61, Fig. 2F). Furthermore, we ran a linear regression model in which the tendency to prioritize the *low mean low variability* condition set was predicted, across participants, by confidence (specifically, the difference in mean confidence between the two conditions, as estimated in those trials where confidence reports were required). Although this model also included the performance difference between the two conditions in the familiarization phase as a covariate, the effect of confidence was significant in the regression (*β* = 0.01, *t* = 3.40, *p* = 0.002).

We ran similar analyses as those just described to test for the effect of response times on priority. First, we correlated, across participants, the difference of median response time in the familiarization phase between *low mean low variability* and *high mean high variability* condition trials with the tendency to prioritize the *low mean low variability* condition set. No significant correlation was found (*r* = − 0.13, *p* = 0.51, Fig. 2G), hinting at response time not having an important role on priority. Next, we built a linear regression model as before, but replacing the difference in performance by the difference in response time between conditions. Even with this last predictor present in the regression, the effect of the difference of confidences was significant (*β* = 0.01, *t* = 3.06, *p* = 0.005). Importantly, in a model where both the difference in performance and the difference in response time between conditions were included, this effect of confidence was still significant (*β* = 0.01, *t* = 3.25, *p* = 0.003). Of note, all analyses in this section were conducted after removing the outlier mentioned in the previous section. In conclusion, confidence had an effect on priority even when accuracy and response times were taken into account.

### Predicting priority from inferred confidence

If, at the start of the test phase, participants prioritized one set over another based on their confidence, this confidence would have been formed when interacting with both sets in the immediately previous familiarization phase. To further support that prioritizing one set over another was related to the confidence associated with the trials in the familiarization phase, we tried to infer confidence for these trials. To do so, we relied on the trials in the last part of the experiment, where confidence was reported. Specifically, for each participant, we fitted a linear regression model where the reported confidence was predicted by condition, response accuracy and response time, given their known relationships with confidence. We then took each participant’s trials for the familiarization phase and used the aforementioned model to infer confidence in these trials where it was not collected. Then, for each participant we calculated the mean inferred confidence over each set in the familiarization phase. The difference between the two sets was finally used to predict which set was prioritized, in a mixed effects logistic regression (fitted in R by maximum likelihood with the *glmer* function, with p-values given by the lmerTest package) that included participants as a random intercept. Critically, this inferred confidence was a significant predictor of priority choices (*β* = 0.02, S.E. = 0.01, *p* = 0.04). As the AIC corrected for small samples (AICc^31^) and the Akaike weights on Table 1 show, the regression model based on inferred confidence (inferred confidence model) explained priority choices better than a null model including only random intercepts for each participant. It also outperformed alternative models where priority choices were predicted by the difference between both sets in terms of accuracy (accuracy model) or response time (response time model).

**Table 1:** For the different models described in the text, the table indicates the AIC corrected for small samples (AICc) and where applicable the difference in AICc compared to the inferred confidence model (ΔAICc), the Akaike weight against the inferred confidence model and the likelihood ratio test relative to the null model.

	AICc	ΔAICc	Akaike weight	Likelihood ratio test
Inferred confidence model	1282.23	–	–	χ^2^(1) = 4.43, *p* = 0.04
Null model	1284.64	2.41	0.23	–
Accuracy model	1285.71	3.48	0.15	χ^2^(1) = 0.94, *p* = 0.33
Response time model	1286.61	4.38	0.10	χ^2^(1) = 0.04, *p* = 0.83

In the inferred confidence model, prioritization choices are predicted from the difference in average inferred confidence between both sets. In the accuracy model, the predictor is the difference in accuracy between both sets. In the response time model, the predictor is the difference in median response time between both sets. All models also included participant ID as a random intercept.

Finally, a model was built where priority choices were predicted by the aforementioned differences in inferred confidence, accuracy and response time (complete model). Although such model fared worse than the model taking only confidence as a predictor, we used it as a baseline for our next analysis, where we built separate regression models by removing one predictor at a time from this full model. We found that removing the difference in inferred confidence resulted in a marginally worse fit compared to the full model (*p* = 0.05, likelihood ratio test), while removing any other predictor caused non-significant improvements (see the table in the supplementary material for detailed reports). This result confirms, again, that priority is affected by confidence more than it is affected by differences in response times or performance.

## Discussion

By dissociating confidence from task performance the present study shows that confidence is specifically associated with how individuals prioritize one task over another. When comparing two experimental conditions associated with different confidence but equal performance, we found that the tendency to prioritize one condition over the other was related to the confidence participants had in their previous decisions in these two conditions. This relationship between priority and confidence was found both across participants by examining inter-individual differences in these two variables, and within participant by examining variations of confidence across trials. Crucially, this relationship could not be explained by the difference of performance between the two conditions. In addition, as confidence was evaluated in the final part of the experiment after the measurement of priority, its role in the determination of priority is unlikely to have resulted from experimenter demand effects. In sum, confidence drives prioritization between two tasks, above and beyond objective performance.

In previous work^22^, confidence has been related both to the prioritization of reporting already-made decisions and to the prioritization of yet-to-complete tasks. However, in the latter context confidence was not collected via reports, but approximated by a task difficulty manipulation which led to different performance levels. This may have raised the concern that performance, rather than confidence itself, was driving priority. The present work addresses this shortcoming by asking for confidence reports and by experimentally dissociating performance from confidence.

While our work supports confidence as a driver of priority, in most situations priority will also be influenced by other factors. Besides obvious determinants like predefined rules, differences in explicit reward^32^ or perceived effort between tasks^33^, the literature also points to past experience of reward with a task^34,35^, familiarity and personal interest in a task^36^, the presence of subgoals within a task^37^, task duration^21^ or difference in deadlines between tasks^38^. Biases may also occur that are not related to the nature of the task themselves, but to personality traits and demographic variables, as it happens in procrastination^39^. Some other biases could relate to the procedure by which task ordering takes place. For instance, factors such as the order of appearance in the to-do list or the similarity between the next task and the current task could also play a role, as elaborated in the next paragraph in the context of our study.

To isolate the specific role of confidence in priority choices we dissociated confidence and perceptual performance by relying on an experimental manipulation of the mean and variability of stimulus evidence^23,26^. As expected, we found a higher confidence for the *low mean low variability* condition when compared to the *high mean high variability* condition, despite equal performance^18,23–25^. Thus, our initial prediction was that individuals would prioritize the *low mean low variability* condition overall. We were surprised to find that this basic prediction was not confirmed, despite clear evidence for the link between confidence and priority in our data. A possible explanation is that priority choices may have been affected by other factors apart from confidence. In some cases, these other biases may have overridden the effect of confidence. In the supplementary material we look into some of these biases. Overall, we found a spatial bias by which participants tended to choose the set whose name was presented on the left and a bias towards the last presented set during the familiarization phase. Would such biases help participants make quick priority choices? Similarly, would they provide an advantage for performance? For instance, in the case of a long to-do list, completing all tasks in a sequential order by starting with the first item (i.e. choosing the left box in our design) could help ensuring that no task is forgotten while limiting the memory demands for knowing which task remains to be done. Choosing the last presented set could also be advantageous in our design, as it limits the number of times a participant has to switch between the different experimental conditions. Although such switches were not between different tasks, each condition involved stimuli with different mean and variability. We know that humans perform better when operating with stimuli that have the same variability as the stimuli they recently operated with^40^. Thus, participants could have chosen to stay within the same condition not to modify their expectations about the distribution of the perceptual stimuli. The supplementary material reports how, also in our experiment, staying within the same condition enhanced performance in the first set of the test phase.

The effect of confidence on priority could be expected to be bigger—and that of the other biases to be lower—if only one of the two sets had to be completed. Indeed, in that scenario, selecting the set associated with the highest confidence would be equivalent to maximizing subjective expected reward, as confidence reflects the subjective probability of being correct. We may thus anticipate that there would be greater incentives for individuals to solely rely on confidence to guide their behavior and to avoid the costly influence of recency or spatial biases in that case. We note that the role of confidence in such selection problems has been studied previously. When individuals have a choice between two conditions of a perceptual task, they prefer to engage in the condition in which their confidence is highest^41^. In the metamemory literature, studies have shown that before a knowledge test, as individuals are given the opportunity to restudy items of their selection, they prefer to restudy items about which they feel less confident^9–11^. In both of these situations, individuals are maximizing subjective expected performance when they engage in the condition associated with high confidence or when they choose to restudy items about which they are less confident (as they should obtain greater benefits in the test by increasing their knowledge for these items). In the aforementioned metamemory tasks, this is made more obvious when considering the existing time limit, either because the duration of study sessions was imposed by the experimenter or because there was a time at which the learnt knowledge was tested. Relative to this prior work, the novelty of the present study is to show that confidence determines priority not only in a task selection problem, but in a task ordering problem where both tasks have to be completed anyway and where time limits could not trigger the effect of confidence. In our context, thus, finding an influence of confidence on prioritization is even more surprising.

The present study provides a new perspective on the relation between retrospective confidence judgments and prospective confidence. Indeed, whereas confidence ratings were collected in a retrospective manner in our study (by asking participants to rate their confidence after each decision), it is interesting to see that such retrospective judgments could be used to predict prioritization choices, which are essentially prospective as they refer to future actions. Prospective confidence refers to how individuals evaluate their future success on specific tasks. For instance, in the meta-memory literature, studies commonly rely on “judgments of learning” by which participants indicate their beliefs that an association learned in the study phase will be recalled accurately in the subsequent test phase. Such judgments have been shown to determine how students allocate their effort to specific items in the study phase^42^. In the domain of perception, direct prospective judgments are not commonly used (but for exceptions see^43,44^). Instead, indirect measures have sometimes been interpreted as reflecting an on-going or prospective estimation of performance, such as the speed with which we point at a response^45^ or the choice to perform one task rather than another^41^.

The relationship between retrospective and prospective confidence has been investigated in several recent studies. For instance, people have been shown to exhibit a similar amount of idiosyncratic over/underconfidence across domains^44^. Also, prospective reports influence subsequent retrospective estimates^43,46^. However, retrospective judgments seem to be more accurate, probably because trial-specific cues can be used^47^. In addition, retrospective confidence is influenced by previous judgments over a longer timescale^44^. Our study contributes to this line of research by providing evidence that retrospective confidence may be used in a prospective manner in prioritization problems.

One key methodological aspect of our study was to infer confidence in the familiarization trials, rather than measuring confidence directly in those trials. The use of confidence in one part of the experiment to predict prioritization in another part is legitimated by the very similar performances in the different parts of the experiment (see supplementary material). Also, response times in all experiment parts were lower for the *low mean low variability* condition, consistent with higher confidence. To go beyond, we also built individual models where confidence was explained by accuracy, response times and condition, and used these models to infer what reported confidence may have been for the familiarization phase trials. Crucially, this inferred confidence could still explain priority choices, and in fact it explained them better than accuracy or response times alone. More generally, this result suggests that relying on a proxy for confidence can provide a helpful leverage for future studies where explicit confidence ratings cannot or should not be obtained from participants.

Our study delves on the role of prospective confidence estimates. Such estimates are typically built from repeated experience with past similar situations. Thus, rather than reflecting confidence on a single decision, prospective confidence is the result of aggregating multiple decisions. Past work has examined how we integrate single decisions to form “global” confidence estimates and how the way those estimates are integrated impacts future decisions^6,48,49^. The current study shows another way in which past experience about a set of perceptual decisions, and more specifically the confidence associated with such decisions, may determine future behavior, in this case related to prioritization.

Finally, although the present study closes a gap opened by previous research, there are many aspects that can still be investigated on the relationship between priority and confidence. In this paragraph we propose that future research could answer different questions related to individual differences, subjective phenomenology and neural correlates of this effect. First, one could investigate how the effect of confidence on priority may differ across individuals. Here, we found that prioritization was influenced by confidence in the conditions, as well as by spatial biases (prioritizing the option presented on the left) or recency biases (prioritizing the last-presented condition). However, as we know that biases and heuristics vary across individuals^50–52^, understanding how individual characteristics (e.g. gender, age or personality traits) may modulate the determinants of prioritization is an open avenue for research. A second future line of work may explore the phenomenology associated with the effect of confidence or biases on prioritization choices. In particular, ascertaining whether participants deliberately implement a prioritization strategy by relying more heavily on one of those factors or whether this weighting occurs implicitly. A related question would be on the phenomenology associated with confidence in a decision. Here, we speculate that confidence makes the task more salient, and that this experienced saliency is what determines the weight of confidence on prioritization choices. More empirical investigations are needed to investigate these issues, including the collection of phenomenological data through direct inquiry to participants, either during the experiments or at the end of them as part of the debriefing. As a third question for follow-up research, we consider the neural correlates of prioritizing based on confidence. Even in a simple experimental situation like ours, this process involves many computational steps, each with potentially different brain regions involved. We know of the key roles of both medial prefrontal cortex^5^ and ventral striatum^6^ in forming global confidence estimates from repeated experience with a task. We also know that the anterolateral prefrontal cortex is crucial when comparing prospective confidence in two tasks in order to engage in the task associated with highest anticipated confidence^53^. A logical continuation would be to extend these investigations to the case of prioritization between two tasks, both of which have to be done.

In conclusion, the present study lends support to confidence having a unique role, independent of performance, in guiding prioritization between forthcoming tasks. This shows another facet of the role of confidence in regulating behavior.

## Supplementary Information

Supplementary Information.

## Data Availability

The experiment code, as well as the datasets generated and analyzed during the current study, are available from the corresponding author on reasonable request.
