# Supplementary material for: Confidence guides priority between forthcoming tasks
Source: Sci Rep. 2021 Sep 15;11:18320. doi: 10.1038/s41598-021-97884-2 (PMC8443637; doi:10.1038/s41598-021-97884-2)
Supplement: Supplementary file 1 — Supplementary Information. [file 41598_2021_97884_MOESM1_ESM.pdf]

CONFIDENCE GUIDES PRIORITY BETWEEN FORTHCOMING TASKS

David Aguilar-Lleyda & Vincent de Gardelle

SUPPLEMENTARY MATERIAL

Correlation of performances among experiment parts

In the main text we showed that performance was not different between conditions (see also Figure 1A) and also that a relationship existed between greater confidence in the *low mean low variability* condition and greater tendency to prioritize *low mean low variability* condition sets (see figures 2D and 2E). However, it is also worth checking that performances among the different parts of the experiment were similar within participants. This is important, since in our study we link performance in the main part of the experiment with confidence in the final trials where it had to be reported. We found that, across participants, performance in the familiarization trials was correlated with performance in the test trials, both for the *high mean high variability* condition (r = 0.82, p < .001) and for the *low mean low variability* condition (r = 0.93, p < .001). Figure S1A illustrates this. We then pooled all the trials of the main part together and ran a correlation with performance on the final trials where confidence had to be rated (see Figure S1B). The correlation was significantly positive for both conditions (*high mean high variability* condition: r = 0.72, p < .001; *low mean low variability* condition: r = 0.83, p < .001).

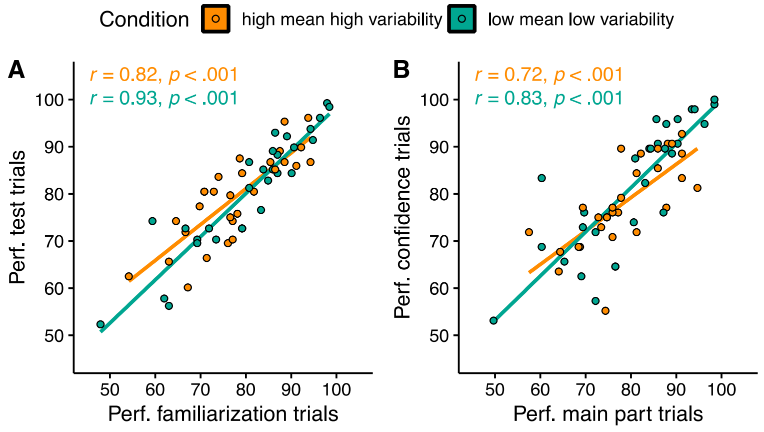

Figure S1. A. Average performance for the familiarization trials against average performance for the test trials. **B.** Average performance for the main part of the experiment (with the familiarization and test trials being pooled together) against average performance for those trials where confidence had to be rated. For A and B, each participant is depicted with two dots, one corresponding to each condition (color-coded). Lines represent linear fits for each condition's data.

Supplementary table for "Predicting priority from inferred confidence"

The main text describes how a complete model was built where prioritization choices were predicted by differences between conditions in inferred confidence, accuracy and response time. This model was used as a baseline to build further models by dropping one predictor at a time. Then, differences were examined between each of these new models and the complete model. Table S1 displays the statistics mentioned in that group of analyses. An AICc increase with respect to the complete model means that the dropped term is an important predictor. Such increase only happened when dropping inferred confidence, further evidencing the role of confidence as a driver of prioritization.

	AICc	ΔAICc	Likelihood ratio test
Complete model	1286.02	-	-
Without inferred confidence	1287.73	1.71	$\chi^2(1)=3.73$, $p=0.05$
Without accuracy	1284.01	-2.01	$\chi^2(1)=0.01$, $p=0.92$
Without response time	1284.24	-1.78	$\chi^2(1)=0.25$, $p=0.62$

Table S1. Taking the complete model described in the main text as a baseline, the table indicates the AICc when each predictor is dropped from the complete model and where applicable the difference in AICc compared to the complete model (ΔAICc) and the likelihood ratio test against the complete model. The complete model had which set was prioritized as dependent variable. The predicting variables were difference in average inferred confidence between both sets (inferred confidence), difference in accuracy between both sets (accuracy) and difference in median response time between both sets (response time). The model also included participant ID as a random intercept.

Choice biases affecting priority

In our experiment, the absence for a general tendency of confidence affecting priority may be due to the presence of choice biases. For some participants, these biases could have influenced priority choices in a stronger way than confidence did. In this section we explore two obvious choice biases. The first one is a tendency to choose the response box occupying a certain side of the screen (left/right) when making their priority choices. The second one is the tendency to choose either the set that was presented first (or last) in the familiarization phase. To see whether we could find general effects of these biases, we built a logistic regression model. The dependent variable was whether the *low mean low variability* condition had been prioritized. The predictors were whether the name associated to the *low mean low variability* condition had occupied the left choice box and whether the *low mean low variability* condition had been presented first. Moreover, we controlled for the effect of performance by including a third predictor: the difference in correct choices in the familiarization phase between the *low mean low variability* and the *high mean high variability* conditions. While this difference in performance had no significant effect (β = 0.04, S.E. = 0.05, p = .34), there was a very significant tendency to choose the left choice box (β = 0.28, S.E. = 0.07, p < .001) and a significant tendency to prioritize the last-presented set (β = 0.17, S.E. = 0.07, p = . 01). Hence, our data points towards the existence of simple choice biases that also affected priority choices.

We also wondered whether prioritizing the last-presented set could bring an advantage in performance. In the discussion of the main text we mention the existence of priming effects for stimuli with similar statistics as those recently experienced. Given that priming, it could be that people performed better in the first-completed set of the test phase (i.e. the prioritized set) if this was of the same condition as the last-presented set of the familiarization phase. To find support for this hypothesis, we took, for each participant, all the first-completed sets of the test phase and calculated average performance, split by whether those sets had belonged to the same or to the other condition as the last-presented set of their respective familiarization phases. A paired t-test gave a significant difference in performance, favoring those first-completed test sets matching in condition the last-presented familiarization sets (difference of 3.10%, t(28) = 2.44, p = .02, 95% CI [-0.06, -0.01], d = 0.45, Bayes factor for difference between conditions = 2.44). Figure S2 graphically depicts this difference.

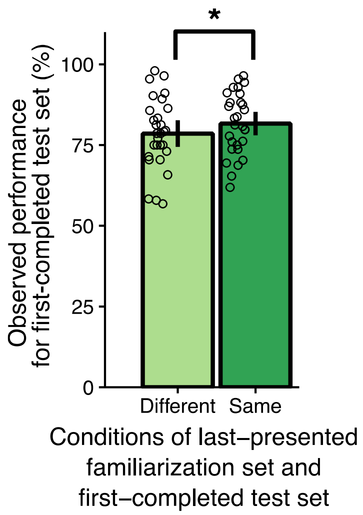

Figure S2. For the first-completed sets of the test phase, observed performance as a function of whether, in that block, the last-presented set of the familiarization phase and the first-completed set of the test phase had belonged to the same or to a different condition. Bars depict across-participant averages and error bars represent 95% confidence intervals. Dots show individual participants. The line connecting the pair of columns is accompanied by a symbol representing the *p* value for the corresponding paired t-test described in the text (n.s.: $p \geq .05$; *: $.05 > p \geq .01$; **: $.01 > p \geq .001$; *** : $p < .001$).

Screenshot of the confidence scale appearing on the confidence trials

The final trials of our experiment included confidence ratings. However, the main text is not accompanied by any graphical representation of the confidence scale that was used for those ratings. Figure S3 below corresponds to an actual screenshot of the screen where a confidence rating had to be made. The question, in French on the figure, can be translated to ‘To what extent are you sure that your choice was correct?’. Participants moved a cursor along the scale and clicked whenever the integer appearing on the left matched their confidence level. On the Figure, the cursor is at a y-position corresponding to a confidence of 78%.

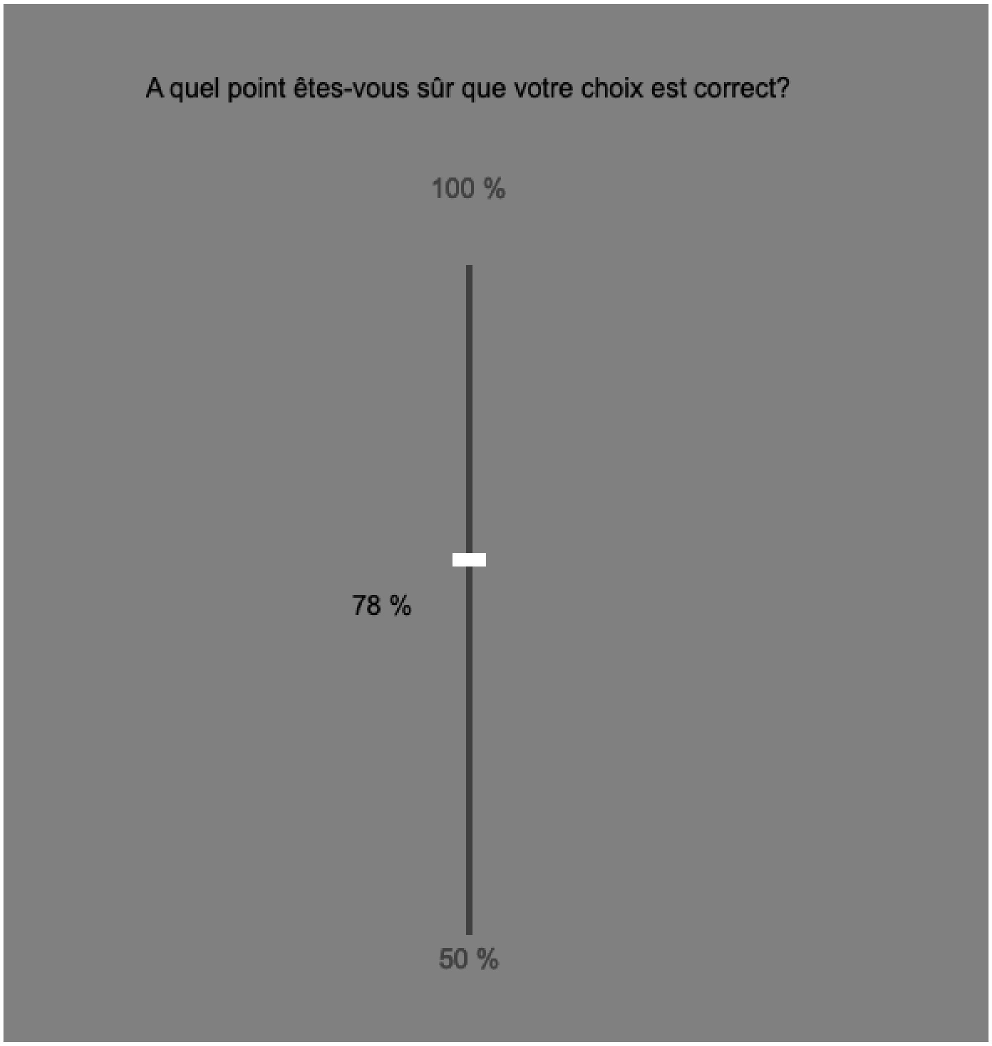

Figure S3. Screenshot of a confidence rating screen. The borders have been cropped so the scale is magnified.

List of animal names used to label experimental sets

Here we present a list of the animal names used to label sets in our experiment. These are the same used in Experiment 4 of Aguilar-Lleyda et al. (2020). Original French names, in capital letters, were those displayed in the experiment. Here they are accompanied by their English translation (between brackets).

ABEILLE (bee)	AGNEAU (lamb)	AIGLE (eagle)
ANTILOPE (antelope)	AUTRUCHE (ostrich)	BABOUIN (baboon)
BALEINE (whale)	BISON (bison)	BUFFLE (buffalo)
CANARD (duck)	CASTOR (beaver)	CHAMEAU (camel)
CHENILLE (caterpillar)	CHEVAL (horse)	CHÈVRE (goat)
CHIEN (dog)	CHOUETTE (barn owl)	CIGOGNE (stork)
COCHON (pig)	COYOTE (coyote)	CRIQUET (cricket)
DAUPHIN (dolphin)	ÉCUREUIL (squirrel)	ÉLÉPHANT (elephant)
ESCARGOT (snail)	FOURMI (ant)	FURET (ferret)
GAZELLE (gazelle)	GIRAFE (giraffe)	GORILLE (gorilla)
GUÉPARD (cheetah)	HAMSTER (hamster)	HERISSON (hedgehog)
HIBOU (owl)	HYENE (hyena)	JAGUAR (jaguar)
LAPIN (rabbit)	LIMACE (slug)	LOUTRE (otter)
LUCIOLE (firefly)	MARMOTTE (groundhog)	MOINEAU (sparrow)
MOUTON (ram)	OTARIE (seal)	PANDA (panda)
PAPILLON (butterfly)	PIGEON (pigeon)	POISSON (fish)
PONEY (pony)	POULE (chicken)	RENARD (fox)
RENNE (reindeer)	REQUIN (shark)	SANGLIER (boar)
SCARABÉE (beetle)	SERPENT (snake)	SINGE (ape)
SOURIS (mouse)	TAUREAU (bull)	TIGRE (tiger)
TORTUE (turtle)	VACHE (cow)	VAUTOUR (vulture)
ZEBRE (zebra)		
